# Relative abundance and the fate of human rotavirus in wastewater during treatment processes: identification of potential infectious rotavirus in the final effluents and receiving aquatic milieu in Durban area, South Africa

**DOI:** 10.1007/s10661-024-12888-5

**Published:** 2024-07-18

**Authors:** Cornelius Arome Omatola, Tosin Abiola Olasehinde, Ademola Olufolahan Olaniran

**Affiliations:** 1 Discipline of Microbiology, School of Life Sciences, University of Kwazulu-Natal, Westville, Durban, Kwazulu-Natal Province South Africa; 2 Nutrition and Toxicology Division, Food Technology Department, Federal Institute of Industrial Research, Oshodi, Lagos Nigeria

**Keywords:** Wastewater treatment, River, Real-time PCR, ICC-qPCR, Rotavirus

## Abstract

The occurrence and persistence of rotaviruses in raw and treated wastewater and their discharge into rivers represent a significant health risk for humans and animals, worldwide. In this study, samples were collected monthly from each of the four Durban wastewater treatment plants (DWWTPs) and receiving rivers for a period of 3 months. Rotavirus was quantified by real-time quantitative PCR (RT-qPCR), and viability was assessed using integrated cell culture (ICC)-qPCR. Rotavirus was detected consistently in 100% of influent wastewaters (mean concentration range, 4.36–4.46 log_10_ genome equivalent (GE) copies/L) and final effluent samples of three DWWTPs (range, 3.35–3.61 log_10_ GE copies/L). Overall, 94% (45/48) of the wastewater analyzed and 95% (20/21) of the associated river water samples were positive for rotavirus (range, 2.04–6.77 log_10_ GE copies/L). The activated sludge process with 0.10–0.43 log_10_ reduction values (LRV) only moderately reduced the viral loads. Similarly, one of the DWWTPs that operated the biofilter modality produced 0.20 LRV. Though the additional treatment with chlorine produced higher LRV (range, 0.31–0.53) than the corresponding activated sludge or biofilter process, the difference in viral removals was not significant (*p* > 0.05). The equivalent treatment efficiencies of the four DWWTPs varied from 19 to 43% decay in the population of rotavirus. Further, infectious rotavirus ranging from 66.67 to 100%, 50 to 100%, and 66.67 to 100% were detected in the post-activated sludge, final effluents, and river water samples, respectively. In conclusion, the findings of infectious rotavirus in both the final effluents and associated rivers represent an infection risk for humans or animals during contact. Thus, close monitoring for rotavirus and risk assessment studies under distinct exposure scenarios may further shed light on the health-related risks associated with water recovery and reuse in urban settings.

**Supplementary Information:**

The online version contains supplementary material available at 10.1007/s10661-024-12888-5.

## Introduction

As of April 2021, more than 56% of South Africa’s wastewater treatment plants were in poor or critical conditions while 75% of 910 municipalities could not achieve up to 50% compliance with the minimum microbiological standard for effluent river discharges (Kretzmann et al., 2021). The large increase in human population coupled with the climate crisis and the resultant increase in shortage of dependable water supplies have led to emerging global demand for reclaimed or recycled water from treated wastewater for recreational activities, irrigation in agriculture, industrial use, and a source of raw water for drinking water production (Prado et al., 2019; Kretzmann et al., 2021). In recent years, climate change in South Africa has intensified due to the increase in national average temperatures and a decrease in rainfall patterns (USAID, 2022). Climate change heavily impacts water security, with more recurrent drought and water shortages leading to water scarcity in many parts of the country (USAID, 2022). In the country, millions of people experience myriads of the economic, health, and recreational impacts of viral pollution instigated by the daily disposal of billions of liters of poorly treated or completely untreated municipal sewage into the aquatic milieu (Kretzmann et al., 2021). In the Eastern Cape province of South Africa, approximately 42% viral positivity rate (concentration range, 1.9 × 10^3^ to 1.2 × 10^5^ genome copies/L) has been detected in the final effluents of wastewater treatment plants (Osuolale & Okoh, 2017). Elsewhere in the country, human adenoviruses and enteroviruses were detected in 85% and 100% of surface water samples, respectively (Lin & Singh, 2015). Further, the potential for infectivity of viruses transmitted through environmental routes had been confirmed in 100% of water samples (Lin & Singh, 2015), suggesting that the waterborne route represents a significant exposure pathway for human enteric viruses. The current municipal wastewater treatment modality though can eliminate most microbial pathogens, including bacteria and protozoa, does not efficiently do the same for waterborne enteric viruses transmitted via fecal–oral or respiratory routes (Kitajima et al., 2014; Prado et al., 2019). Evidently, run-offs with enteric pathogens from the sewerage systems, which directly entered the water environments, have been linked to waterborne viral gastroenteritis outbreaks in South Africa (Ukhahlamba District Municipality Addendum (UDMA), 2008; Sekwadi et al., 2018). Elsewhere, the occurrence of waterborne rotavirus gastroenteritis outbreaks has been documented in connection with sewage pollution in different epidemiological surveillance studies (World Health Organization (WHO), 2018; He et al., 2009; Hafliger et al., 2000). Therefore, sewage pollution constitutes a potential threat of incidences of infectious viral diseases.

The bacteriological profiles (e.g., total coliforms and enterococci) used as tracking indicators for microbial contamination of water and the treated wastewater effluents are poorly correlated with the presence of viruses due to the diminutive physical size and greater resistance of viruses to wastewater treatment procedures as compared to bacteria (Qiu et al., 2018). Among the gastrointestinal viruses that are common in treated municipal wastewater and aquatic ecosystems, norovirus and rotavirus occurrence are more significant because of their capacity for environmental persistence and viral roles in infectious diarrhea (Qiu et al., 2018; Huang et al., 2022). The health risk associated with the presence of the latter virus in aquatic ecosystems is more in poor socioeconomic countries where rotavirus vaccine coverage is low and opportunities for viral transmission are wide-ranging (Corpuz et al., 2020).

Rotavirus can occur naturally in sewage and environmental waters, but the continuous discharge of the former through anthropogenic activities remains the major gateway to viral introduction into the aquatic milieu (Omatola & Olaniran, 2022a). Humans and animals, when exposed to rotavirus in sewage or contaminated water even at a low dose (< 100 viral particles), stand a risk of being infected because of high viral infectivity (Qiu et al., 2018). Human contamination may occur directly by fecal–oral route or indirectly through the consumption of untreated/partially treated water, contaminated raw food, or filter feeders that have concentrated rotavirus in the edible tissues (Corpuz et al., 2020; Omatola & Olaniran, 2022a). Worldwide, rotavirus is responsible for a significant burden of diarrheal disease in children, though public health interventions targeting immunization and hygiene and the development of advanced tertiary wastewater treatment modalities (reverse osmosis, ultrafiltration, oxidation, and chemical clarification) have shown some potential to reduce environmental spread of the virus and water-related gastroenteritis (Prado et al., 2019; Bennett et al., 2021). The triple-layered structure of rotavirus, high fecal shedding, and their resistance during wastewater treatment processes facilitate the environmental diffusion (Ibrahim et al., 2016) and persistence as well as potentiate viral pathogenicity by facilitating fecal–oral transmission and efficient transport into the intestinal lumen (Omatola & Olaniran, 2022a). Globally, rotavirus remains the major cause of acute infectious gastroenteritis in early childhood, accounting for approximately 258 million morbidity cases and 128,000 diarrheic child deaths per year (Troeger et al., 2018). More recently, a 3-decade observational trend study of the global burden of disease identified rotavirus as the leading etiology of diarrheal deaths in 2019. In particular, countries in Africa, South Asia, and Oceania bear the brunt of the diarrheal deaths (Du et al., 2022). Diarrhoeal diseases are currently ranked the third major cause of childhood mortality in South African children, with rotavirus playing a dominant etiologic role in diarrheal morbidity and rates of hospital admission (UNICEF South Africa, 2018; Asowata et al., 2018).

Wastewater has been recognized as one of the most concentrated sources of rotavirus in the environment, and contamination of other aquatic matrixes is likely when there is damage to the sanitary network or when raw and/ or inadequately treated wastewater is discharged into the environment (Lizasoain et al., 2018). Thus, it is not surprising that the occurrence of rotavirus is frequently reported in association with wastewater, groundwater, freshwater sources, filter feeders (e.g., oysters and mussels), and vegetables garnered from contaminated water (Omatola & Olaniran, 2022a). The potential public health risks associated with wastewater reuse are usually linked to inadequate removal of pathogenic viruses including rotaviruses, which are found in high concentrations in untreated wastewater and are highly infectious to humans (Kitajima et al., 2014). Importantly, viral stability in the aquatic environment implies an increasing need for routine monitoring of rotaviruses in wastewater to ascertain the possible risks that are associated with human exposure and further provide relevant information on the effectiveness of treatment modalities and other intervention strategies (Potgieter et al., 2020).

Recently, there have been emerging reports of rotavirus resistance to chlorine or UV disinfection treatment even with a properly working wastewater treatment system (Kitajima et al., 2014; Rizk et al., 2019; Zhang et al., 2022). Rotavirus occurrence in river water associated with sewage pollution had been documented during a gastroenteritis outbreak investigation on the KwaZulu-Natal Coast (Sekwadi et al., 2018). Thus, the significance of rotavirus in sewage and its efficiency of removal requires investigation to understand the public health-related risks. In South Africa, most epidemiological studies are case-based and only a few environmental surveillance studies (Potgieter et al., 2020; Olaniran et al., 2012; Lin & Singh, 2015) for rotavirus have been reported in some regions/provinces of the country. In the KwaZulu-Natal region, very limited data are available regarding rotavirus occurrence in the sewerage system and water bodies. To our knowledge, there is currently no documented report on rotavirus removals in the sewerage system and potential viral infectivity in the study area. The previous study in the area only documented the virological qualities of some freshwater resources near the Durban area (Olaniran et al., 2012; Lin & Singh, 2015). Therefore, this study assessed rotavirus occurrence and distribution in the sewerage sources and receiving water bodies qualitatively and quantitatively. We also evaluated the efficiency of rotavirus removal/inactivation by the Durban wastewater treatment facilities and further assayed viral potential for infectivity through an integrated in vitro cell culture real-time reverse transcription-quantitative polymerase chain reaction (ICC-RT-qPCR) approach.

## Materials and methods

### Sampling location and sample collection

Wastewater samples were collected from four urban wastewater treatment plants (WWTP) in different locations in the Durban area of KwaZulu-Natal province, South Africa. The Phoenix Wastewater Treatment Plant (PWWTP), Northern Wastewater Treatment Plant (NWWTP), Umbilo Wastewater Treatment Plant (UWWTP), and Isipingo Wastewater Treatment Plant (IWWTP) treat approximately 25–50, 60–70, 23, and 10.98–20 million liters per day of raw sewage, respectively, from the inhabitants of Phoenix, Newlands East, Pinetown, and Umlazi (https://www.boschholdings.co.za/project/northern-and-phoenix-wastewater-treatment/). The population equivalents in the catchment area were 176,989 (PWWTP), 92,792 (NWWTP), 373,086 (UWWTP), and 497,593 (IWWTP). The first three plants operate through the conventional activated sludge treatment modality while the fourth plant utilizes the biological filter for wastewater treatment. All the plants monitored are among the major WWTPs in the KwaZulu-Natal province.

Two (2) liters each of the sewage (influent, primary effluent, post-activated sludge or biofiltered sample, and final effluents) and receiving river water (upstream and downstream of WWTPs) samples were aseptically collected once per month by grab sampling method between August and October 2021. Overall, 69 samples comprising 48 sewage and 21 river water samples were obtained. The characteristics of each sample (place, location, date, and month) were immediately recorded on the sterile plastic bottles before they were transported in portable ice chests to the Microbiology Department of the University of Kwazulu-Natal for processing within 24 h of collection.

### Rotavirus concentration in sewage and river water samples

Samples were clarified by centrifugation (3000 × g, 30 min, 4 °C) using the Beckman Coulter Avanti® J-26 XPI centrifuge. The skimmed milk (SM) flocculation procedure described by Calgua et al. (2008) was adapted for rotavirus concentration. Briefly, a pre-flocculated skimmed milk solution made by dissolving 10 g of skimmed milk powder in 1 L of autoclaved distilled water was pH adjusted with 1N HCL to 3.5. Twenty milliliters of the resulting solution were added to each of the 2 L water samples, which were also pH adjusted to 3.5. Samples were then stirred (8 h, room temperature) followed by flocs precipitation through gravity for a further 8 h. Supernatants were removed such that the sediments were not dislodged, and the final volume was centrifuged (7000 × g, 30 min, 4 °C). The pellets were re-suspended in 2 mL of 1 × phosphate-buffered saline (PBS) after the supernatants were removed, pH adjusted with 1N NaOH to 7.4, and then stored at − 80 °C until assayed.

### Assessment of rotavirus concentration protocol

To assess the efficiency of rotavirus recovery from the skimmed milk flocculation concentration procedures, 2 L of deionized water was spiked with 1 mL of a virus stock suspension (5.5 × 10^4^ genome equivalent (GE) copies/L) and one unseeded sample was used as a procedural negative control. Both the seeded and unseeded samples were run in triplicates using the real-time reverse transcription quantitative PCR (RT-qPCR) after the concentration procedure described above. Rotavirus recovery rate (%) was determined by comparing the proportion of virus in the concentrate to the viral loads of those after the concentration procedure (Pang et al., 2012; Qiu et al., 2015).

### Extraction of viral nucleic acid

Viral dsRNA was column extracted from 1 mL of concentrated samples, using the commercial geneJET total RNA Purification kit (Thermo Fisher Scientific Inc., USA). The eluted RNA in 50 µL of RNase-free water was used in the retro-transcription stage. The purification procedure was in accordance with the kit’s manufacturer specifications.

### cDNA synthesis and quantitative PCR (qPCR) for rotavirus detection

In the reverse-transcription stage, 5 µL of extracted RNA heated at 95 °C for 5 min was chilled on ice for 3 min prior to conversion to the complementary DNA (cDNA) using the iScriptTM Advanced cDNA Synthesis Kit (Bio-Rad, USA). The 20 µL final reaction volume contained 5 × iScript reaction mix (i.e., deoxyribonucleoside triphosphates, RNase inhibitor, RT buffer, oligo-dT, and random hexamer primers), Moloney murine leukemia virus (MMLV) reverse transcriptase, nuclease-free water, and template RNA. The reaction cocktail was incubated (25 °C for 5 min, 46 °C for 20 min, and 95 °C for 1 min) to produce cDNA copies of the genomic RNA.

The qPCR was executed on a CFX96TM Real-Time Optics System with a C1000 Touch Thermal Cycler in a 20 µL total reaction volume containing 10 µL SYBR Green qPCR master mix (Bio-Rad, USA), 2 µL of template DNA, 1 µL each of forward and reverse primers, and 6.0 µL nuclease-free water. Primer pairs (5′-ACCATCTACACATGACCCTC-3′ and 3′-GGTCACATAACGCCCC-5′) with an expected amplicon size of 87 were applied for the detection of a highly conserved gene sequence encoding non-structural protein 3 (NSP3) of group A rotavirus (Pang et al., 2004). Three negative controls and positive control (cDNA of Helix EliteTM inactivated rotavirus molecular standard) with 10^−2^, 10^−4^, and 10^−6^ dilutions were employed for each run. All samples were run in triplicates. Additional quality control measures included the use of separate rooms for sample preparation, reagent preparation, and amplicon visualization. The qPCR condition was set at 95 °C for 3 min for initial denaturation followed by 40 cycles of 95 °C for 15 s, 56 °C for 30 s, and 72 °C for 30 s, corresponding to denaturation, annealing, and extension, respectively. The melting curve analyses (conditions, 0 s at 95 °C, 120 s at 65 °C, and 0 s at 95 °C with ramp rate at 0.5 °C per second) were employed for each run, and agarose gel electrophoresis with a 2% strength was further used to confirm the amplicon size of positive real-time PCR analysis (Supplementary Fig. 1). An external standard curve (10^5^, 10^4^, 10^3^, 10^2^, 10^1^, and 10^0^ copies per reaction) was established with a serially tenfold diluted cDNA of the SA-11 rotavirus strain (Microbiologics, USA) (Supplementary Fig. 2). Using the standard curve and the cycle threshold (Ct) values, the viral concentration in the samples expressed as log10 genome equivalent (GE) copies per liter were determined. To assess for possible presence of chemical inhibitors of RT-PCR, a preliminary investigation was carried out on representative inflow sewage samples (two from each treatment plant). The RNA extracts from the concentrated samples (diluted tenfold) were retested by NSP3-specific RT-PCR, and the results were compared with corresponding qPCR results of undiluted inflow samples.

### Integrated cell culture real-time quantitative PCR (ICC-qPCR)

The ICC-qPCR was carried out to assess the virus’ viability in the pre-chlorinated sewage, post-chlorinated effluent, and associated river water samples that previously indicated positive signals on qPCR. The ICC-qPCR protocol used was adapted from the methods of Rigotto et al. (2010) and Prado et al. (2019). Caco-2 cell line (American Type Culture Collection, US; accession number: HTB-37) which shows the distinctive features of human small intestinal enterocytes and is suited for experiments reconnoitering the effects of rotavirus (Bruno et al., 2022) was used to assay viral potential for infectivity. Caco-2 cells in 6-well microplates were grown under 5% CO_2_ atmospheric pressure at 37 °C in a Dulbecco’s Minimum Essential Medium (DMEM) enhanced with heat-inactivated fetal bovine serum (FBS) (10%), non-essential amino acids (1%), antibiotics (penicillin–streptomycin) (1%), and L-glutamine (4mmol). After 48 h, the cell culture medium containing FBS was removed, and the cell monolayer was washed twice with PBS. One milliliter of each sample inoculated onto confluent Caco-2 cell monolayer in each well was allowed to adsorb to the cell monolayer for 60 min at 37 °C by rocking every 15 min. Inoculums not adsorbed to the cell layer were removed by washing with 1 mL PBS. The cell monolayers were overlaid with 5 mL of DMEM and 2.5% FBS and then incubated at 37 °C under 5% CO_2_ for 24 h. Negative (cultured cell monolayer with DMEN) and positive controls (Simian rotavirus SA11 cultivated in MA104 cell lines) were included in the analysis. After incubation, the supernatants were removed from the microplates, and cell layers were washed three times with PBS. Each plate was subjected to three freeze–thaw cycles for cell lyses and virus release. From the recovered supernatant, 0.5 ml of cell lysate was obtained for nucleic acid extraction and PCR assay.

### Data analysis

The log_10_ reduction value (LRV) achieved by the wastewater treatment process was calculated by the equation (Hmaied et al., 2015):

$$\text{LRV}={\text{log}}_{10}\left(\text{rotavirus influent concentration}/\text{rotavirus effluent concentration}\right)$$

The quantifications and recoveries data set were summarized using descriptive statistics. Some quantification data were summarized with boxes and whiskers. The data sets were analyzed with statistical software packages for social sciences (SPSS) version 16 for Windows (SPSS Inc., USA). The analysis of variance (ANOVA) was used as test statistics to assess the level of statistical significance in rotavirus loads across sampling months of influent and effluent river discharges as well as across sewage receiving rivers. In addition, the non-parametric Mann–Whitney test was performed to assess the level of statistical significance of the LRV between PS and post-chlorination steps. Further, the chi-square test was used to evaluate the statistical significance of cultured positive samples between post-activated sludge/biofilter and post-chlorination. For all test statistics, probabilities (*p*) values < 0.05 were set as the level of statistical significance.

## Results

### Prevalence of rotavirus in raw and at different stages of treated wastewater samples

**Fig. 1:**
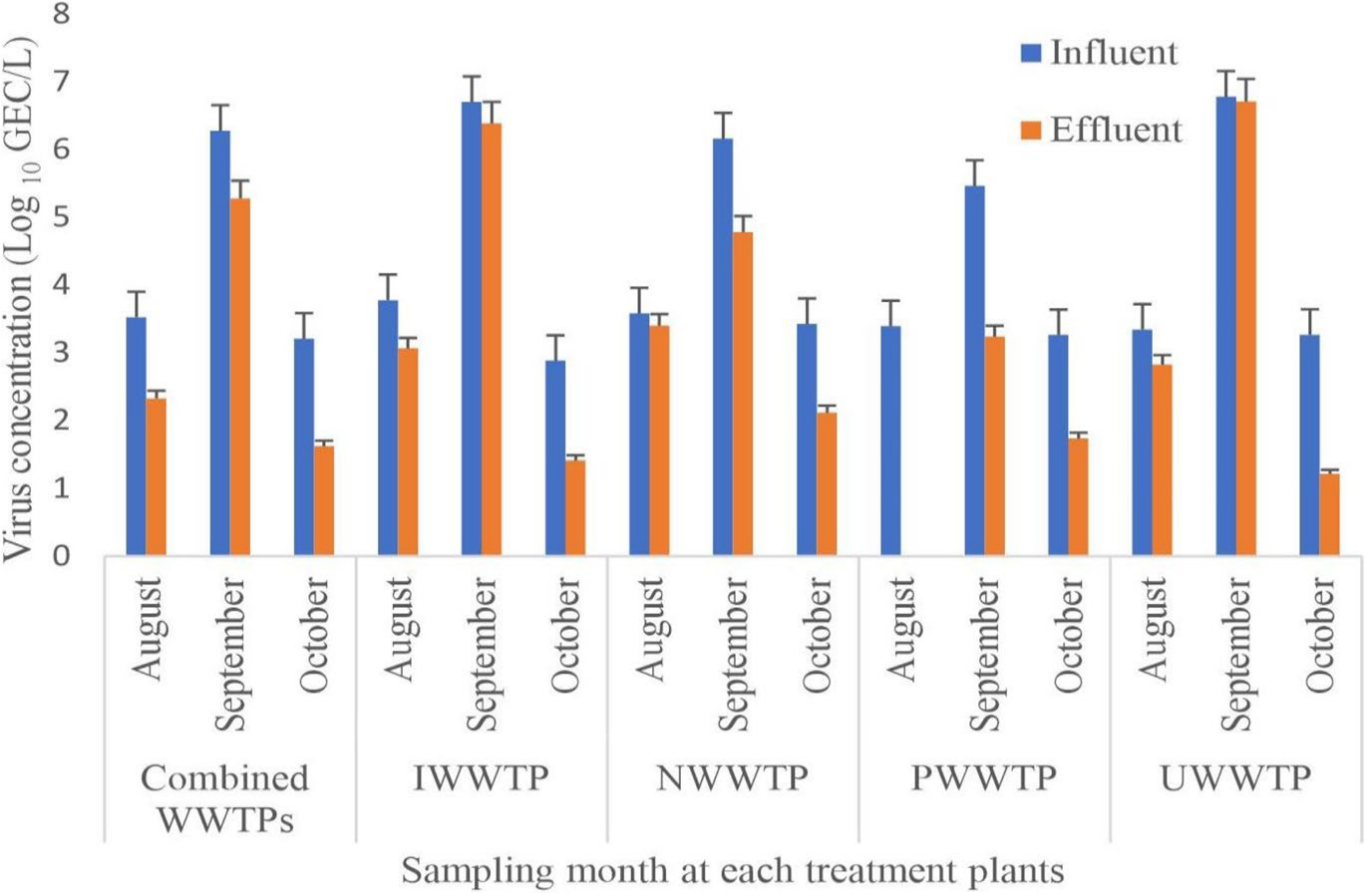
Variations in rotavirus concentration in influent and final effluents during the sampling period. Note: PS = primary sedimentation, ST = secondary treatment, AS = activated sludge, BF = biofiltration, NWWTP = Northern Wastewater Treatment Plant, PWWTP = Phoenix Wastewater Treatment Plant, UWWTP = Umbilo Wastewater Treatment Plant, IWWTP = Isipingo Wastewater Treatment Plant

Rotavirus recovery efficiency by the skimmed milk flocculation concentration protocol was estimated at between 86 and 90% of the spiked viral RNA. A 100% rotavirus detection was observed in all influents of four treatment plants. Similarly, rotavirus was detected in all downstream treatment steps of NWWTP, UWWTP, and IWWTP except PWWTP where 66.7% virus positivity was observed (Table 1). The mean concentrations for the influents ranged from 4.36 log_10_ GE copies/L (Min) (PWWTP) to 4.46 log_10_ GE copies/L (Max) (UWWTP). Overall, 94% (45/48) of the sewage and 95% (20/21) of the receiving river water samples were positive for rotavirus. Generally, the mean viral loads decreased progressively across the downstream treatment steps of the four wastewater treatment plants while the percentage positivity remained unchanged except for PWWTP. For instance, a mean rotavirus reduction of 1.04, 1.88, and 0.88 log_10_ GE copies/L was produced by NWWTP, PWWTP, and UWWTP, respectively, post-chlorination step (Table 1). Figure 1 shows the viral concentration in the influents and final effluents river discharges for each treatment plant during the sampling period. The viral loads in the influent drawn from all the WWTPs were higher in September than in other months with concentrations ranging from 5.46 log_10_ GE copies/L (PWWTP) to 6.78 log_10_ GE copies/L (UWWTP). Similarly, high effluent river discharges ranging from 3.24 log_10_ GE copies/L (PWWTP) to 6.71 log_10_ GE copies/L (UWWTP) were detected in the same month. The results of ANOVA revealed statistical significance differences between viral loads and sampling months for both influents (*F* = 74.1, *p* = 0.001) and effluents (*F* = 12.4, *p* = 0.004). Our results of the RT-PCR inhibition test based on RNA extract dilution as described elsewhere (Prado et al., 2019) generally indicated a decrease in rotavirus concentration in diluted sewage samples (Supplementary Fig. 3).

**Table 1:** Rotavirus detection and quantification in raw and at different stages of treated wastewater samples

Treatment plant	Influent		PS		ST (AS or BF)		Post chlorination	
	*n* (%)	Mean ± StDev	*n* (%)	Mean ± StDev	*n* (%)	Mean ± StDev	*n* (%)	Mean ± StDev
NWWTP	3 (100)	4.39 ± 1.25	3 (100)	3.96 ± 0.13	3 (100)	3.72 ± 0.98	3 (100)	3.35 ± 0.10
PWWTP	3 (100)	4.36 ± 1.10	2 (66.67)	3.44 ± 0.81	2 (66.67)	3.01 ± 0.45	2 (66.67)	2.48 ± 0.75
UWWTP	3 (100)	4.46 ± 1.64	3 (100)	4.09 ± 0.87	3 (100)	3.99 ± 0.97	3 (100)	3.58 ± 0.30
IWWTP	3 (100)	4.45 ± 0.63	3 (100)	4.09 ± 0.84	3 (100)	3.92 ± 0.83	3 (100)	3.61 ± 0.07

*PS* primary sedimentation, *ST* secondary treatment, *AS* activated sludge, *BF* biofiltration, *n* number of positive samples, *StDev* standard deviation, *NWWTP* Northern Wastewater Treatment Plant, *PWWTP* Phoenix Wastewater Treatment Plant, *UWWTP* Umbilo Wastewater Treatment Plant, *IWWTP* Isipingo Wastewater Treatment Plant. The mean values were determined from the log number of rotavirus concentrations (log_10_ GE copies/L). The mean and standard deviation were for positive samples only

### Rotavirus removal during the wastewater treatment processes

**Fig. 2:**
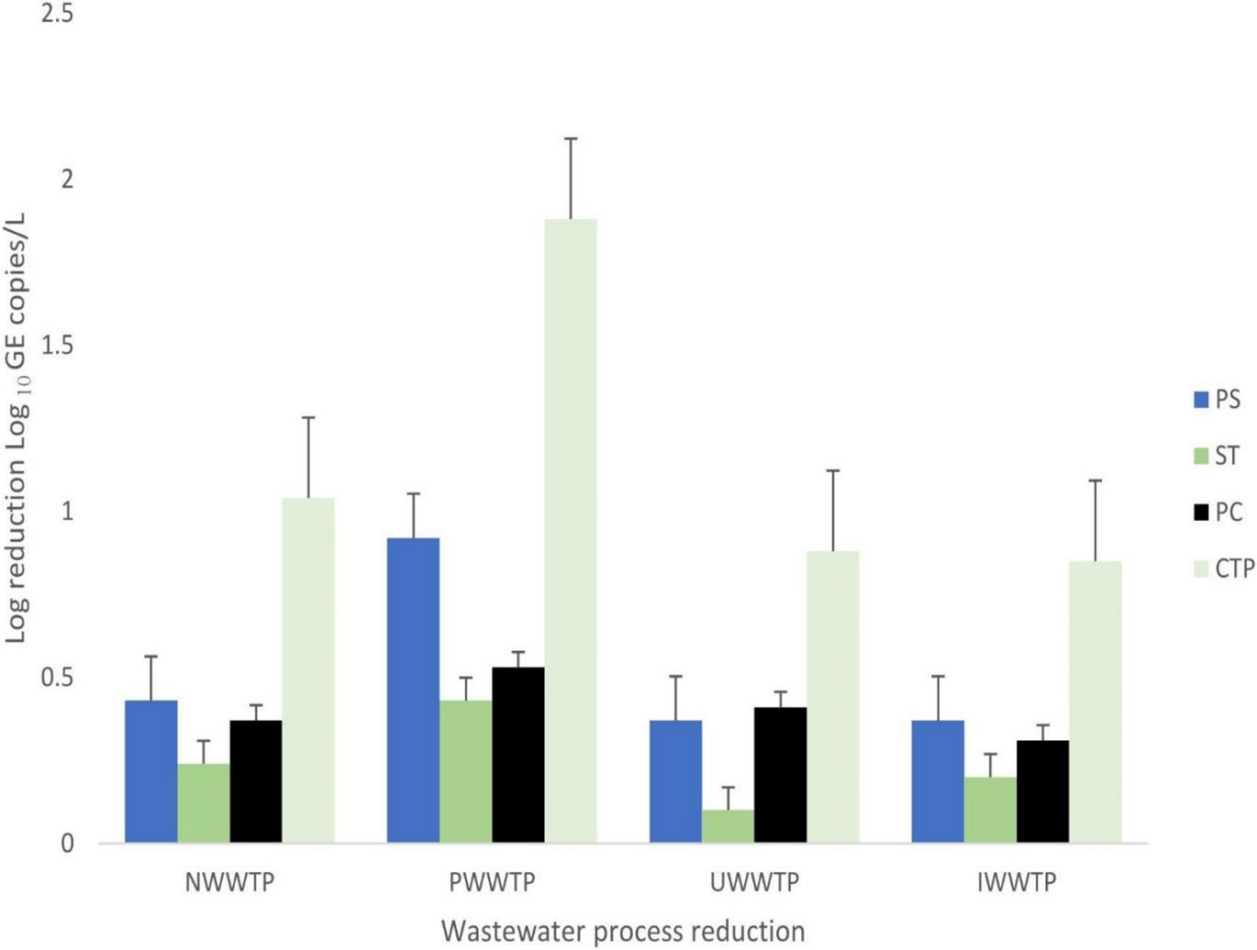
Log reduction of rotavirus after each stage of wastewater treatment. Note: PS = primary sedimentation, ST = secondary treatment, PC = post chlorine, StDev = standard deviation, NWWTP = Northern Wastewater Treatment Plant, PWWTP = Phoenix Wastewater Treatment Plant, UWWTP = Umbilo Wastewater Treatment Plant, IWWTP = Isipingo Wastewater Treatment Plant, CTP combined treatment processes. The plots and error bars depict mean values and standard deviation, respectively

The mean log reduction values (LRV) achieved for each treatment step in rotavirus removal for samples that tested positive for rotavirus are presented in Fig. 2. The activated sludge process with LRV ranging from 0.10 log_10_ GE copies/L (Min) (UWWTP) to 0.43 log_10_ GE copies/L (Max) (PWWTP), only moderately reduced the viral loads. A similar moderate viral decay was observed for IWWTP (LRV; 0.20 log_10_ GE copies/L), which employs the biofiltration treatment modality. Additional treatment with chlorine produced higher LRV (range, 0.31 to 0.53 log_10_ GE copies/L) than the corresponding activated sludge (AS) or bio-filtration (BF) process. However, the test statistics showed no significant difference in viral removals between PS (primary sedimentation) or ST (secondary treatment) and TT (tertiary treatment or post-chlorine) (*p* > 0.05). Overall, the LRV achieved by the four treatment plants varied from 0.85 log_10_ GE copies/L (corresponding to IWWTP that operated the trickling filter technology) to 1.88 log_10_ GE copies/L for PWWTP which employs the activated sludge process. The equivalent treatment efficiencies of the four Durban WWTP facilities varied from 19 to 43% decay in the population of rotavirus.

### Prevalence of rotavirus in Durban rivers receiving treated effluents

**Fig. 3:**
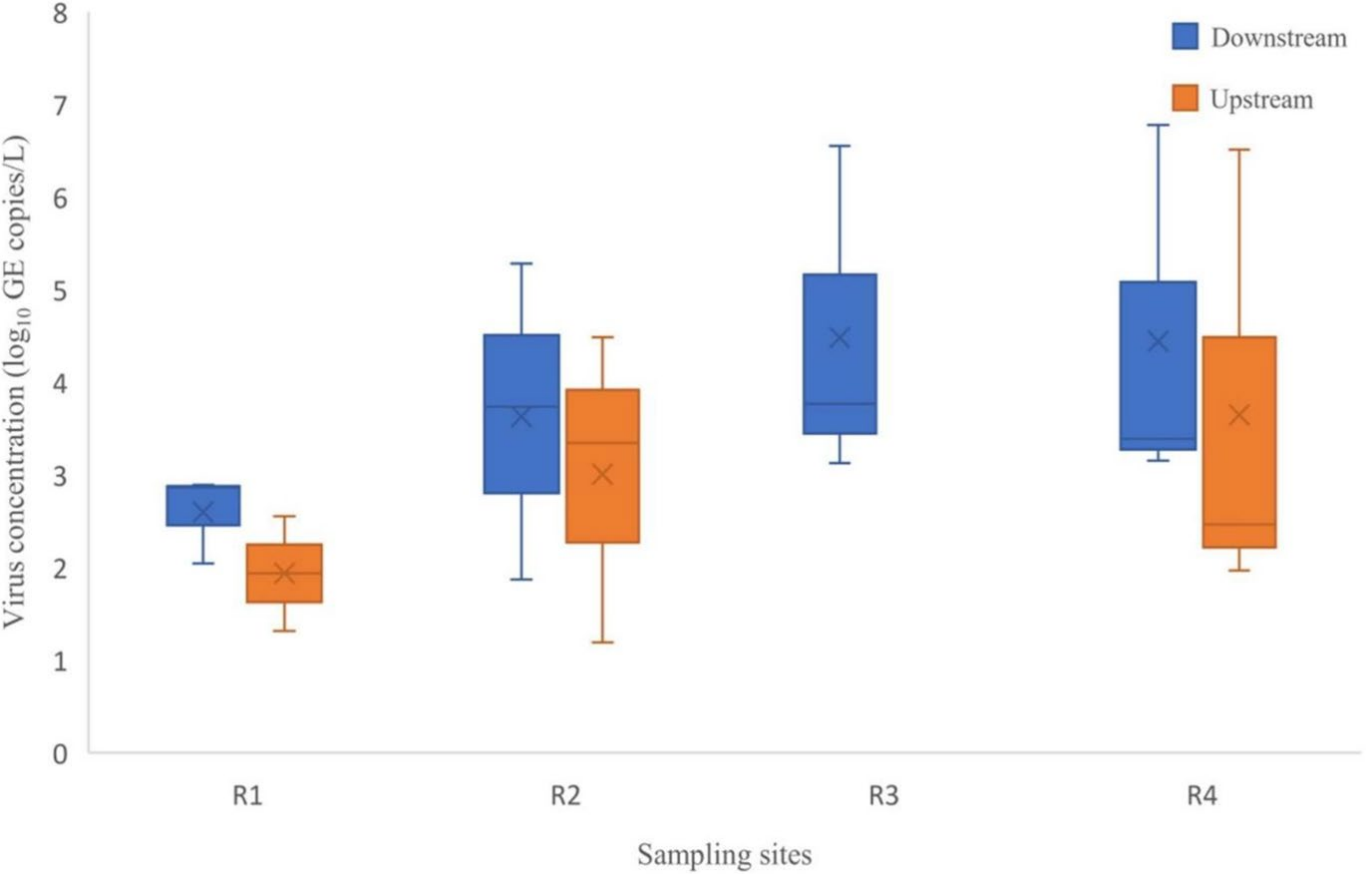
Box plots showing detected rotavirus concentration (log_10_ GE copies/L) at different sites of four receiving Durban rivers. Note: R1 = Phoenix river, R2 = Umgeni river, R3 = Isipingo river, and R4 = Umbilo river

**Fig. 4:**
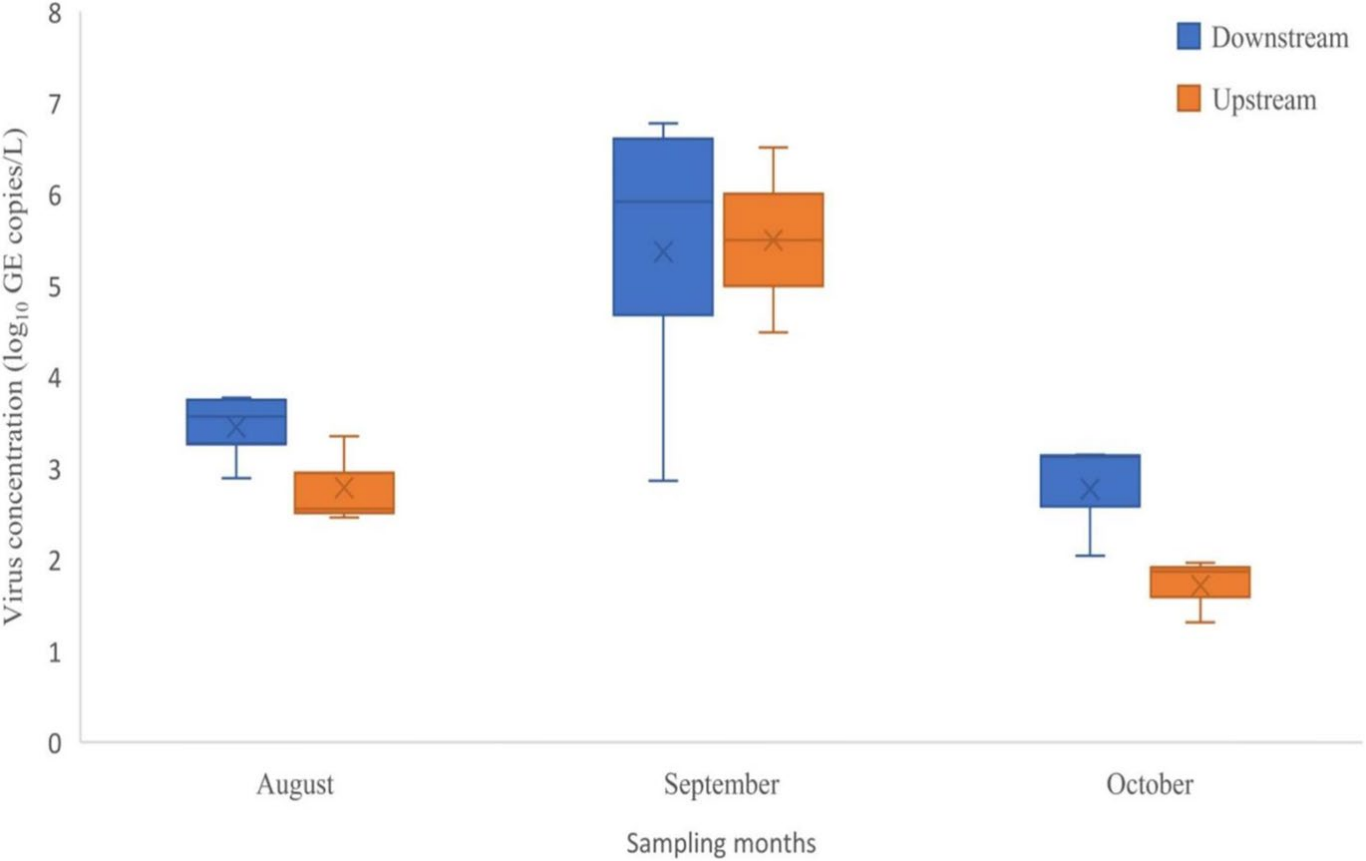
Box plots showing variation in rotavirus concentration (log_10_ GE copies/L) upstream and downstream of the receiving rivers during the sampling period

Figure 3 depicts rotavirus loads in Durban rivers—R1 (Phoenix River), R2 (Umgeni River), R3 (Isipingo River), and R4 (Umbilo River) receiving sewage effluents from the PWWTP, NWWTP, IWWTP, and UWWTP, respectively. Even though test statistics show no statistical difference in rotavirus concentration across sampling rivers (*p* > 0.05), rotavirus loads tend to vary across the sampling sites with the R4 that is linked to the UWWTP indicating a higher level of viral pollution. Figure 4 shows the total viral concentration in downstream and upstream river water samples during the sampling period. High viral loads ranging from 1.87 to 6.77 log_10_ GE copies/L were detected in downstream river samples compared to 1.19–6.51 log_10_ GE copies/L of the upstream. Similar to observation in wastewater treatment plants, significantly high rotavirus concentration was observed during September in both the downstream (*F* = 4.8, *p* = 0.04) and upstream (*F* = 12.0, *p* = 0.02) rivers. Generally, rates of rotavirus detection during the sampling period coincide with trends of viral loads in effluent discharges.

### Prevalence of viable rotavirus in post-activated, reclaimed water, and Durban rivers receiving treated effluents

**Fig. 5:**
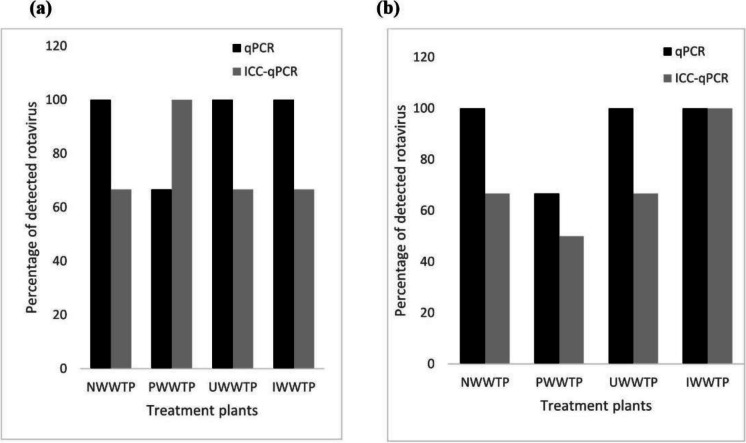
Percentage of viable virus detected by ICC-qPCR in post-activated sludge/biofilter (**a**) and reclaimed water samples (**b**) for each treatment plant compared with the corresponding qPCR values

**Fig. 6:**
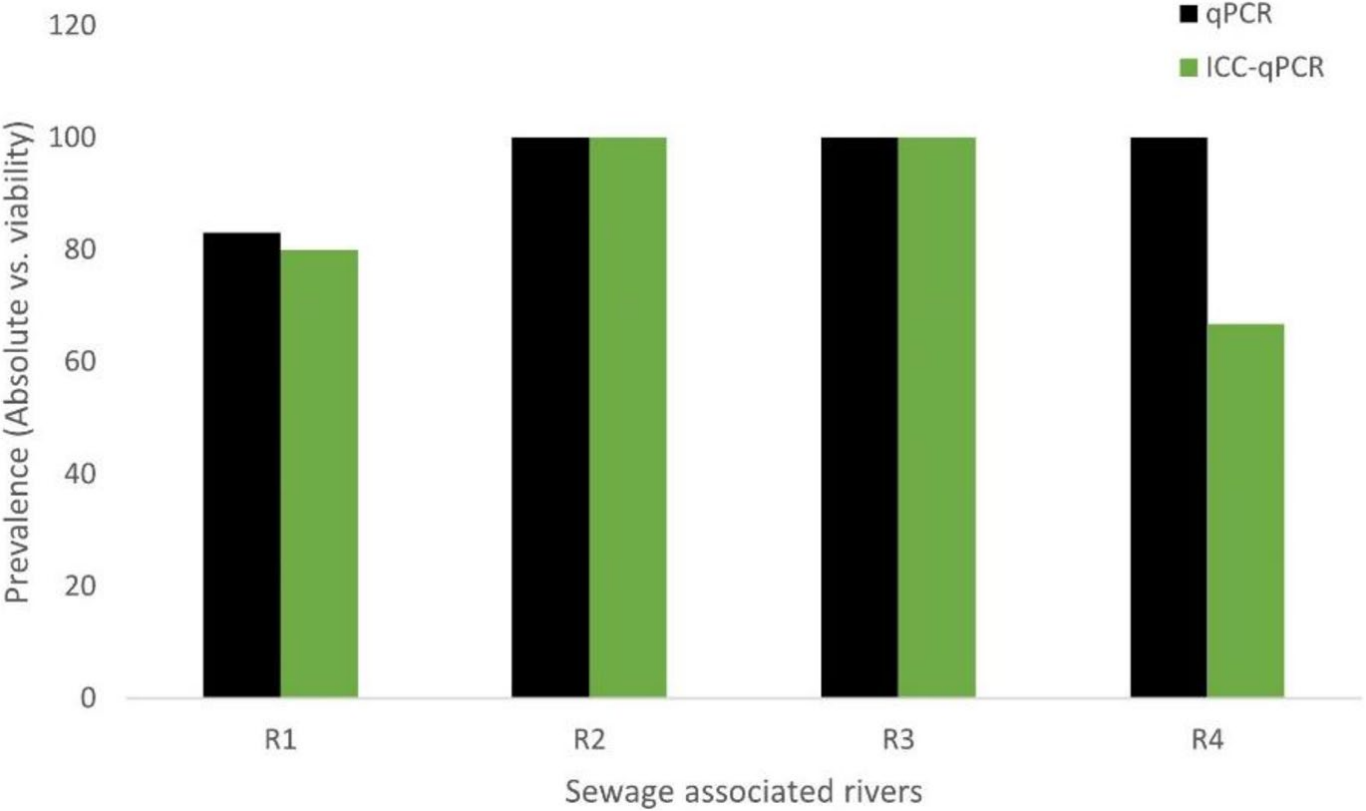
Percentage of viable virus detected by ICC-qPCR in Durban rivers compared with the corresponding qPCR values. Note: R1 = Phoenix river, R2 = Umgeni river, R3 = Isipingo river, and R4 = Umbilo river

The use of ICC-qPCR in the study detected viable rotavirus which ranged from 50 (PWWTP) to 100% (IWWTP) in the final effluents post-chlorine treatment (Fig. 5). However, a chi-square analysis of the difference in the number of viable rotaviruses between post-activated sludge/biofilter and the effluent river discharges of each wastewater treatment plant did not reveal any level of statistical significance in treatment reduction between both processes (*p* > 0.05). In the sewage-associated rivers, Phoenix, Umgeni, Isipingo, and Umbilo (Fig. 6), potentially infectious rotavirus was detected in 80% (4/5), 100% (6/6), 100% (3/3), and 66.67% (4/6), respectively of the cultured samples.

## Discussion

The occurrence of rotavirus in wastewater, relative viral stability in the environment, and the occasional discharge of inadequately treated effluents into aquatic reservoirs have made wastewater-based epidemiologic surveillance an emerging topic owing to the rising need for water reuse on a global scale. This study focused on rotavirus contamination of wastewater and their receiving rivers used for recreational activities, irrigation of agricultural crops, industrial applications, fish farming, and, in some cases, sources of raw water for drinking water production. The skimmed milk flocculation concentration protocol employed in the study produced 86–90% efficiency of rotavirus recovery, which is comparable with the 95% viral recovery reported by Calgua et al. (2013) but higher than the rates (52%; 25.6–30.7%) previously reported by Calgua et al. (2008) and Gonzales-Gustavson et al. (2017) who applied similar method for concentrating rotavirus and other enteric viruses from sewage and environmental waters. The percentage recovery in the study confirms the efficacy of the low-cost skimmed milk flocculation method integrated with qPCR quantification and further suggests its applicability for rotavirus studies in low-resource laboratory settings. Contrary to the reports of Prado et al. (2019), our inhibition test did not indicate an interference of chemical inhibitors with the RT-PCR reactions, an observation likely explaining higher viral recovery in the undiluted samples compared to the diluted ones. Previously, differences in sample composition, physicochemical properties, and handling procedures were shown to influence inhibitors that can be concentrated and, ultimately, viral recovery rates (Haramoto et al., 2018). Thus, the results of our inhibition test may not entirely preclude the presence of inhibitors considering the complexity of the sample matrix and the fact that higher viral loads may be possible in such samples in the absence of inhibitors.

In the current study, relatively high rotavirus concentration was detected in the influents and all samples drawn from all downstream treatment steps. This finding reflects the common presence of rotavirus in raw sewage and the tendency for persistence despite wastewater treatment processes. The 100% of detected rotavirus positivity rate in all influents of WWTP has also been previously reported in Canada (Qiu et al., 2015), Brazil (Prado et al., 2019), and Tunisia (Hmaied et al., 2015). The viral abundance in final effluents of all treatment plants (range, 2.48–3.61 log_10_ genome copies) implies that rotavirus particles are discharged into the receiving natural watercourse essentially used as drinking water, for agricultural irrigation, environmental enhancement, or recreation. In all of the final effluents investigated with ICC-qPCR, up to 73% of infectious rotavirus was detected, suggesting a potential infection risk in connection with wastewater reuse or their dispersion into the receiving aquatic milieu in the Durban area because of high viral infectiousness which has been documented (Kitajima et al., 2014) and the ease with which contaminated natural watercourse can amplify chances of communal spread in a highly susceptible population of children or immunosuppressed individuals (Omatola & Olaniran, 2022a). As a remedy to the widespread findings of high viral concentration in finally treated water in our study and the inconsistent correlation of viral presence with current sanitary bacterial indicators that have been observed elsewhere (Qiu et al., 2018; Lizasoain et al., 2018), there is the need for additional scrutiny to further ensure virologic safety of effluent river discharges.

Despite that the mode of operation in the primary treatment steps of the four plants is similar, the viral log reductions in primary sedimentation effluents of the three treatment plants that operated the activated sludge process were more compared with the biofilter-based plants. The reason for this observation is not clear. Though other forces of interaction including surface charge properties between virus particles, suspended organic solids, and differences in instrument design might have driven the difference. However, a future study that could incorporate the evaluation of the aforementioned characteristics may better shed light on these discrepancies. Further, the ability of the primary sedimentation process to cause viral reduction as observed in those plants has been previously observed by Melnick (1986) in a full-scale primary settling of raw sewage where an estimated 50% virus reduction was documented, with attribution of the process reduction to viral-fecal aggregation and gravity precipitation.

Even though the removal of rotaviruses by the conventional activated sludge process has been well documented (Prado et al., 2019; Lizasoain et al., 2018; Qiu et al., 2015), only limited data are available regarding the removal efficiency by the trickling filter treatment process (Kitajima et al., 2014). In South Africa, this is the first documented report on rotavirus removal by the trickling filter wastewater treatment process, to the best of our knowledge. Assessing the WWTP process existing in the country is imperative to guarantee viral removal from wastewater and produce final effluent of good quality. In both WWTPs utilizing the conventional activated sludge process (NWWTP, UWWTP, and PWWTP) and biological trickling filter technology (IWWTP) studied, only a moderate level of rotavirus removals was produced. A similar partial rotavirus removal from activated sludge and trickling filtered samples has been reported in 1-year monitoring of sewage samples from two independent WWTPs in Southern Arizona (Kitajima et al., 2014), reflecting the inefficiency of both types of treatment modality and equal comparability in viral removals. The mean log reductions (0.10 to 0.43) achieved by the activated sludge process in the study are corroborated by recent data (0.37 to 1.39) from a Brazilian study of four WWTPs, which also indicated moderate rotavirus removals (Prado et al., 2019). Further, findings from a pooled study of wastewater reclamation and reuse systems have indicated 0.20 to 1.53 log reductions for rotavirus in an activated sludge process (Sano et al., 2016), which is similar to our findings and equally suggests the need for continuous monitoring. Consistent with the low efficiency (19%) of rotavirus removal by the trickling plant, Rizk et al. (2019) in Egypt previously reported 29.4% by a WWTP that uses the same plant operation and chemical treatment strategy. The inefficiency of rotavirus removals in the biological activation sludge or trickling filter stage could be attributed in part to the operational conditions of WWTPs and physicochemical parameters of sewage, which have been documented (Sidhu et al., 2017).

In the current study, viral decay was observed during all the treatment steps, though the comparative analysis of log reduction values pre- and post-chlorination stages did not reveal significant removals of rotavirus, an indication likely pointing to chlorine resistance or evasion of the chlorine treatment. The total log reductions (range, 0.85–1.88) obtained in the four WWTPs post-chlorination are lower than the viral LRV of six recommended by the World Health Organization for well-performing wastewater treatment systems (World Health Organization (WHO), 2006). The inefficiency of the plant in removing rotavirus could be attributed to the molecular structure and small physical size of rotavirus which easily aggregate with organic material in sewage (Lizasoain et al., 2018); hence, they are shielded from inactivation by chlorine and are detected in final effluent despite the treatment. Contrary to our findings, up to 5.8 log reduction of rotavirus had been reported in a more advanced WWTP membrane bioreactor system post-chlorination (Sano et al., 2016; Hmaeid et al., 2015), a difference that was attributed to the high viral adsorptive affinity to the membrane of the bioreactor-based treatment platform. Nevertheless, a study by Qiu et al. (2015) has documented a lower mean log reduction of 0.08 for rotavirus at the full-scale post-chlorination step. Furthermore, the overall viral removal efficiencies of 19–43% achieved by the four WWTPs in the Durban area after all downstream treatments indicate that each treatment facility may be dispersing not less than 53% of the virus into the Durban rivers. This conforms with studies by Kitajima et al. (2014) and Rizk et al. (2019) in other parts of the world which indicated that 20–80% of enteric virus removal could be achieved by properly working wastewater treatment systems.

The findings of up to 6.77 and 6.51 log_10_ GE copies/L of rotavirus in the upstream and downstream rivers, respectively, are suggestive of high viral pollution and a potential health risk, given the low viral infectious dose, capacity for long-term persistence in aquatic milieu, and efficient transmission of rotavirus via the aquatic pathways (Omatola & Olaniran, 2022a). In addition to the inefficiently treated effluent river discharges influencing viral pollution, informal settlements in the area leading to pollution whereby wastes are dumped, without any control, into rivers and coastal environments may have contributed to the high viral loads. The finding of infectious rotavirus in 85% of sampled rivers supports a World Health Report (2011) which posited that rotavirus up to 100 particles is common in raw water globally. The facts that these rivers are used for a myriad of activities including irrigation, recreation, industrial, and domestic indicate an urgent need to prioritize safe wastewater discharge and continuous genomic monitoring for rotavirus, in the end-use water to generate early warning signals to forestall any future waterborne gastroenteritis outbreaks, which had been observed previously in the KwaZulu-Natal province (Asowata et al., 2018).

The high concentration of rotavirus in sewage and concomitantly in receiving rivers during September may reflect high fecal shedding from symptomatic and/or asymptomatic carriers in the area during the study period. Although seasonality was not investigated in the current study, the predominance of rotavirus occurrence among the South African pediatric population in winter months (June–August) has been documented severally in the literature (Steele et al., 2003; Asowata et al., 2018). Although September presented with a peak of viral loads across the sampling matrix, the low viral loads observed in August may be due to a chance, considering that grab sampling methods which provide a snapshot of the virus concentration were employed rather than a proper time series investigation. Notwithstanding, September corresponds to early spring in South Africa during which a secondary peak of rotavirus gastroenteritis had been previously observed in the area (Steele et al., 2003). Though meteorological indicators (https://world-weather.info/forecast/south_africa/durban/August-October2021) did indicate close average precipitation between August and September (3 days vs. 5 days), prevailing host and viral factors that have been shown to cause seasonal fluctuations of most rotavirus seasons in the African continent (Omatola & Olaniran, 2022b) might have driven the difference.

In this study, we realize one major drawback of direct quantitative PCR which is its inability to discriminate inactivated or non-infectious viruses from viable viruses (Qiu et al., 2018), which could lead to an overestimation of the health-related risks posed by the treated final effluent reuse or contact with sewage receiving rivers. Hence, the cell culture-based approach, which is the only method currently available for detecting infectious rotavirus, was integrated with qPCR because of the increased sensitivity and ability to ascertain viral viability (Qiu et al., 2015; Prado et al., 2019). Generally, there was a decline in the percentage of viable viruses, results that were expected owing to the impact of the treatment processes on viral viability. Surprisingly, we found an increase in the number of potentially infectious viruses in one of the treatment plants (IWWTP) which corresponded with the absolute qPCR results. This finding is comparable with the previous observation of Qiu et al. (2015) and could be attributed to a sampling effect. Again, in another plant (PWWTP), one of the activated sludge samples which presented with low signals on qPCR was confirmed positive on ICC-qPCR, supporting a greater sensitivity of the latter method relative to the former. The exclusion of the sample from positive qPCR results due to weak signal and the eventual confirmation as viable virus may have influenced the greater percentage of samples indicating viability on ICC-qPCR. Our observation may be further supported by the fact that the cell culture procedure improves the molecular detection of rotaviruses by increasing the titer, thereby enhancing the RT-qPCR amplification signal (Qiu et al., 2018; Omatola & Olaniran, 2022a). Nevertheless, a significant percentage of rotavirus remains viable after both post-activated sludge/biofilter and chlorine treatment, suggesting partial inactivation and equally ruling out the possibility of overestimation of the associated public health risk of viral pollution in the study.

The current study has certain limitations. Firstly, the use of the skimmed milk flocculation concentration procedures could have inactivated the less stable rotavirus genotypes resulting in their possible non-detection on ICC-qPCR. Thus, spiking the samples with stock rotavirus followed by the ICC-RT-qPCR detection modality after the skimmed milk flocculation concentration procedures may further shed on the potential effect of the concentration protocol on viral viability. Secondly, the current study was carried out in one geographical region of South Africa. Consequently, findings from the study may not be generalizable to the entire country. Thirdly, the short duration of surveillance and absence of seasonality consideration owing to funding constraints may have precluded some of the analyses related to the influence of seasonal parameters on the changing trend of rotavirus excretion rate in the population. Ultimately, these could have reduced the in-depth understanding of secondary virologic pollution drivers of surface waters. Notwithstanding, the findings of potentially infectious rotaviruses in both sample sources (sewage and associated rivers) showed that the virus may have survived the municipal sewage downstream treatment processes and rotavirus particles are probably being discharged with treated effluents into the receiving rivers. Thus, in addition to the conventional treatment modalities being used in the area, it is suggested to also implement modifications in the disinfection process workflow and/or use of more advanced wastewater treatment plants membrane bioreactor system to decrease the viral load being discharged into the aquatic bodies. The quantitative data generated in the study provided proof-of-concept and baseline information for informed policy decisions by the relevant agencies on the need for the inclusion of routine wastewater-based surveillance for rotaviruses to generate early warnings and equally regulate effluent discharges.

## Conclusion

The current study showed that data on the occurrence and quantification of rotaviruses in pre-chlorinated and finally treated effluent river discharges could be beneficial to regulatory agencies in the water industry as tracking indicators of water pollution sources. The finding of potentially infectious rotavirus in the post-chlorinated wastewater and the associated aquatic ecosystems is an indication of infection risks and further justifies the WHO inclusion of rotaviruses among major reference pathogens in water predicated on the ability for long persistence in water supplies and the associated potential for infectivity (World Health Organization (WHO), 2022). Thus, increasing attention should be given to the contamination of waters by sewage which remains an important source of rotaviruses causing waterborne and water-related diseases in humans worldwide. Further, the current study shows that monitoring the impact of current rotavirus vaccination on the disease and assessment of the efficiency of rotavirus removal from wastewater will require the continuous evaluation of sewage sources and receiving watersheds for rotavirus to generate information that can be used to inform vaccine efficacy, highlight the possibility of transmission to the human population, and also provide an early warning system for incipient outbreaks of enterically transmitted rotaviruses from different people irrespective of their symptoms in the Durban area. Further studies to understand why rotaviruses persist despite the wastewater treatment process including mechanisms of inactivation and any probable interaction of rotavirus with aquatic microbiomes to check for the possibility of protective hosts are warranted. In addition, exploring novel wastewater treatment strategies such as membrane bioreactor technology may improve rotavirus removal efficiency to reach effluent of good quality.

### Supplementary Information

Below is the link to the electronic supplementary material.

Supplementary Material 1: Supplementary Figure 1. The real-time RT-PCR products stained with ethidium bromide on agarose gel.

Supplementary Material 2: Supplementary Figure 2. A standard curve showing the cycle threshold (Ct) value at the indicated template copy number.

Supplementary Material 3: Supplementary Figure 3. Comparative analysis of rotavirus recovery between undiluted and diluted inflow sewage samples.

## Data Availability

Datasets from this study is available on request.
