# Supplementary material for: Relative abundance and the fate of human rotavirus in wastewater during treatment processes: identification of potential infectious rotavirus in the final effluents and receiving aquatic milieu in Durban area, South Africa
Source: Environ Monit Assess. 2024 Jul 18;196(8):746. doi: 10.1007/s10661-024-12888-5 (PMC11258059; doi:10.1007/s10661-024-12888-5)
Supplement: Supplementary file 1 — Supplementary Material 1: Supplementary Figure 1. The real-time RT-PCR products stained with ethidium bromide on agarose gel. [file 10661_2024_12888_MOESM1_ESM.docx]

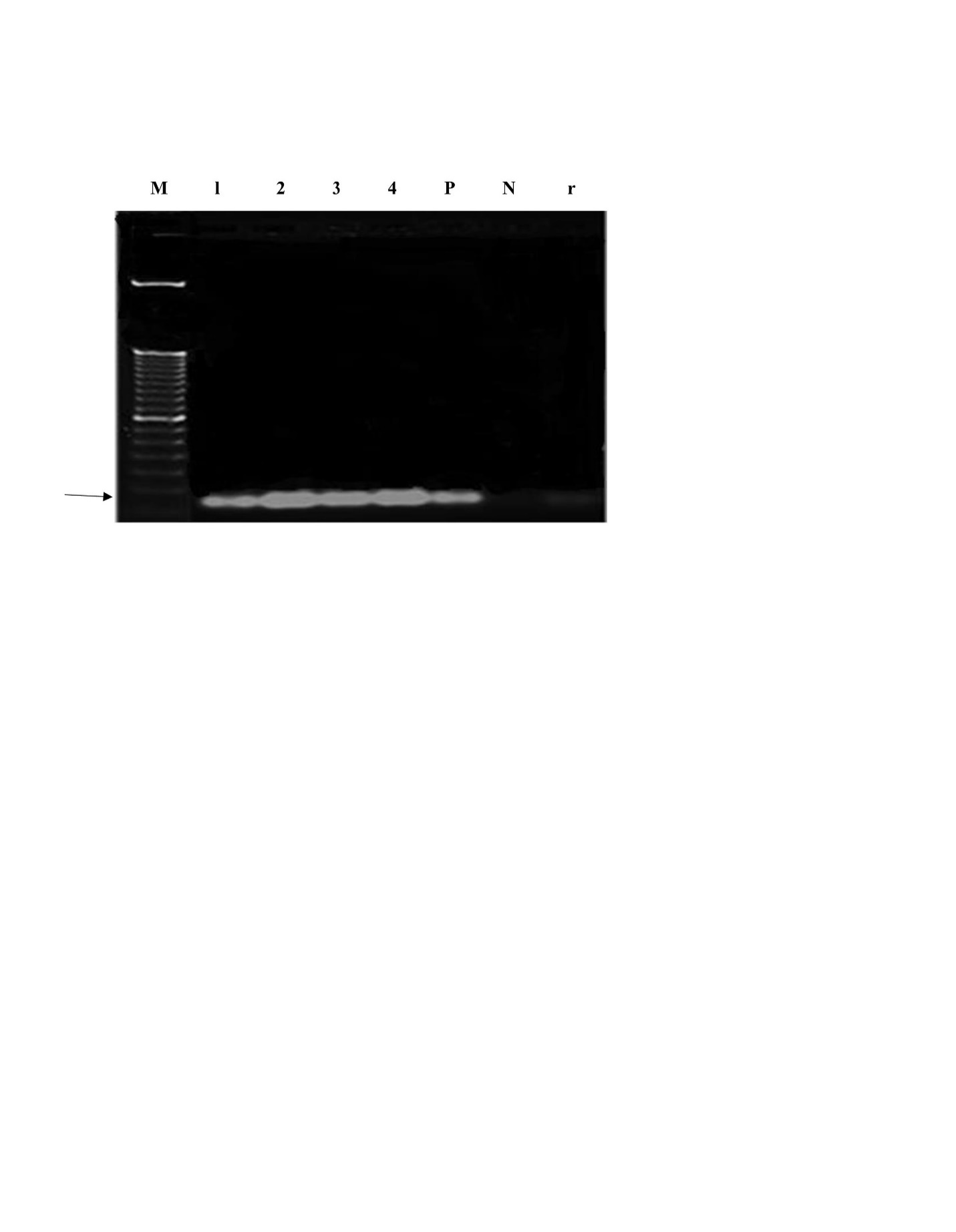


**Supplementary Fig. 1** The real-time RT-PCR products stained with ethidium bromide on an agarose gel. M: 50 bp DNA ladder; Numbers (1-4) indicate individual samples; (P) Positive control reaction; (N) negative control; (r) re-amplified sample. Arrow designates the expected location of the rotavirus NSP3 gene (87 bp) band.
