# Supplementary material for: Relative abundance and the fate of human rotavirus in wastewater during treatment processes: identification of potential infectious rotavirus in the final effluents and receiving aquatic milieu in Durban area, South Africa
Source: Environ Monit Assess. 2024 Jul 18;196(8):746. doi: 10.1007/s10661-024-12888-5 (PMC11258059; doi:10.1007/s10661-024-12888-5)
Supplement: Supplementary file 2 — Supplementary Material 2: Supplementary Figure 2. A standard curve showing the cycle threshold (Ct) value at the indicated template copy number. [file 10661_2024_12888_MOESM2_ESM.docx]

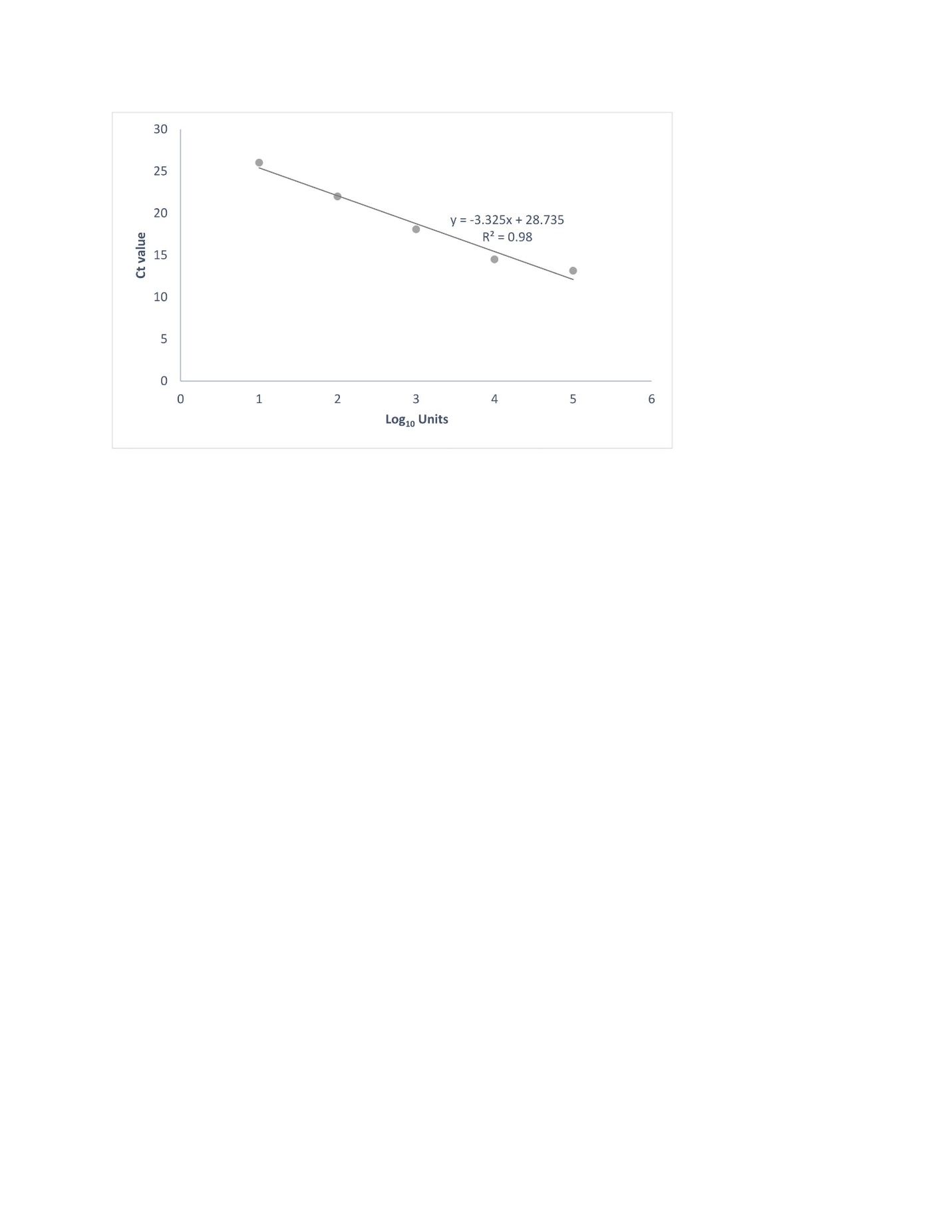


**Supplementary Fig. 2** Standard curve showing the cycle threshold (Ct) value at indicated template copy number.
