# Supplementary material for: Relative abundance and the fate of human rotavirus in wastewater during treatment processes: identification of potential infectious rotavirus in the final effluents and receiving aquatic milieu in Durban area, South Africa
Source: Environ Monit Assess. 2024 Jul 18;196(8):746. doi: 10.1007/s10661-024-12888-5 (PMC11258059; doi:10.1007/s10661-024-12888-5)
Supplement: Supplementary file 3 — Supplementary Material 3: Supplementary Figure 3. Comparative analysis of rotavirus recovery between undiluted and diluted inflow sewage samples. [file 10661_2024_12888_MOESM3_ESM.docx]

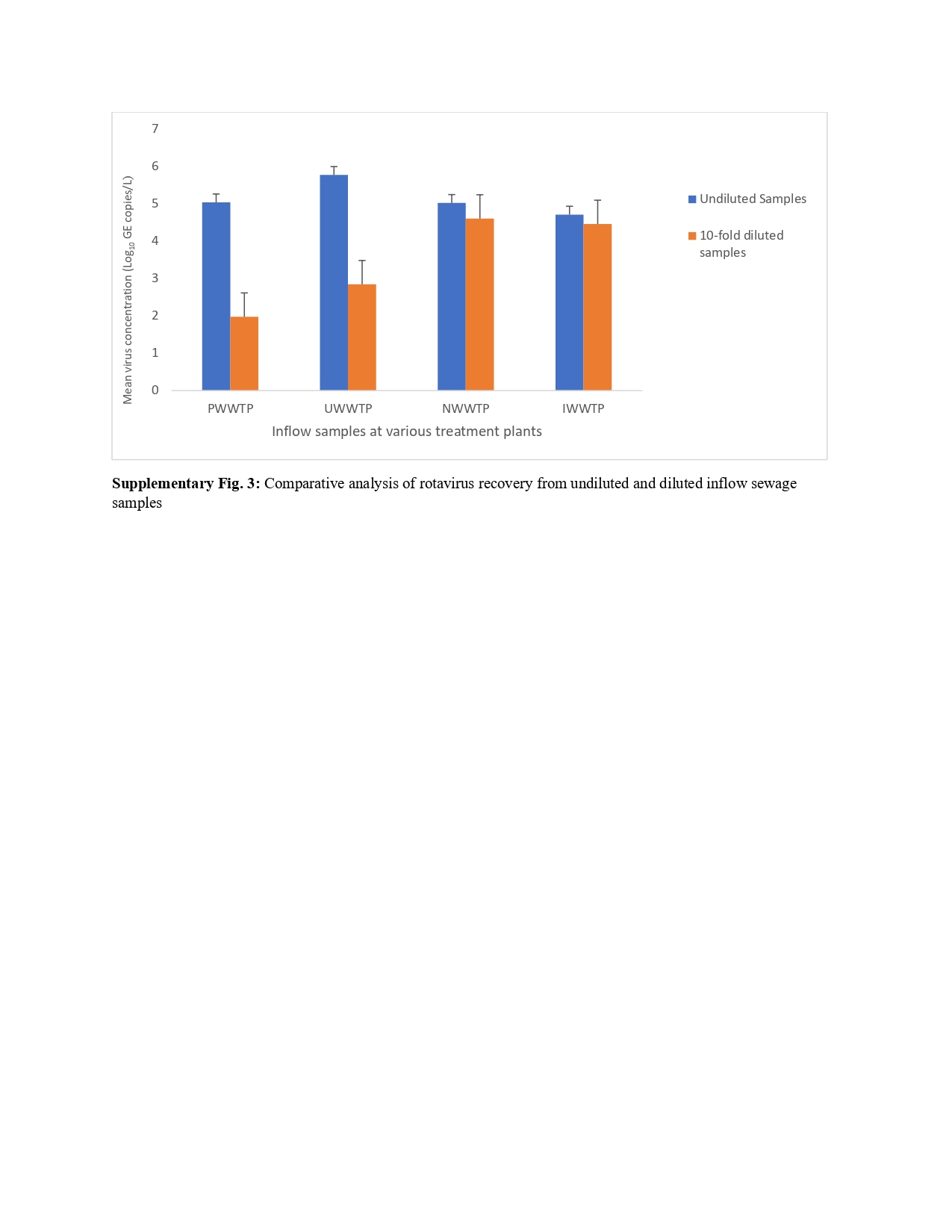


**Supplementary Fig. 3:** Comparative analysis of rotavirus recovery between undiluted and diluted inflow sewage samples
